# Study Profile of the Iwate PGS Assessment and Risk Communication (PARC) Study

**DOI:** 10.2188/jea.JE20250078

**Published:** 2026-02-05

**Authors:** Akiko Yoshida, Tomoharu Tokutomi, Nobuhiro Suzumori, Akimune Fukushima, Yukiko Toya, Hideki Ohmomo, Kozo Tanno, Yoichi Sutoh, Yuka Kotozaki, Tsuyoshi Hachiya, Kazuki Kumada, Hisaaki Kudo, Atsushi Hasegawa, Mika Sakurai-Yageta, Akira Narita, Yohei Hamanaka, Satoshi Nagaie, Soichi Ogishima, Fuji Nagami, Yayoi Otsuka-Yamasaki, Shohei Komaki, Shiori Minabe, Koichi Asahi, Ryujin Endo, Yasushi Ishigaki, Masayuki Yamamoto, Atsushi Shimizu, Makoto Sasaki

**Affiliations:** 1 Iwate Tohoku Medical Megabank Organization, Disaster Reconstruction Center, Iwate Medical University, Iwate, Japan; 2 School of Medicine, Iwate Medical University, Iwate, Japan; 3 Institute for Biomedical Sciences, Iwate Medical University, Iwate, Japan; 4 Tohoku Medical Megabank Organization, Tohoku University, Miyagi, Japan; 5 Graduate School of Medicine, Tohoku University, Miyagi, Japan; 6 School of Nursing, Iwate Medical University, Iwate, Japan

**Keywords:** ischemic stroke, genome cohort, polygenic score, risk communication

## Abstract

**Background:**

The potential impacts of polygenic scores (PGS) on health-behavior changes are not fully understood. The Iwate PGS Assessment and Risk Communication Study aims to investigate the effects of reporting PGS-based risk for ischemic stroke on health behaviors.

**Methods:**

Participants wishing to know their PGS-based ischemic stroke risk were recruited from health checkup venues for workers in Iwate Prefecture in 2023. Health checkup data, biospecimens, and questionnaire responses were collected for biochemical testing, genotyping, and storage in the Tohoku Medical Megabank integrated biobank. The risk was calculated using an integrative PGS model for East Asians. Participants were randomly assigned to two groups, and one group received their risk report as the intervention group. The impacts of the risk notification will be investigated in follow-up surveys.

**Results:**

Of 3,599 workers, 2,088 participated in the study (consent rate: 58.0%). The demographic profile of the eligible 2,083 participants was as follows: 80.7% males, and dominance of participants aged 18–29 years (25.2%), in their 30’s (25.3%), and in their 40’s (24.7%). Two hundred participants (9.7%) had a risk of 1.0 as the reference; 57 (2.7%), 927 (44.7%), and 888 (42.9%) participants had 2.1–3.4-, 1.4–1.9-, and <1.0-fold that risk, respectively.

**Conclusion:**

We collected health information and biospecimens from over 2,000 workers and disclosed the PGS-based ischemic stroke risk. Behavioral effects will be evaluated 1 year after disclosure, with follow-up until 2030. As Japan’s first large-scale PGS risk communication study, it will provide initial insights for implementing PGS in personalized preventive medicine.

## INTRODUCTION

Stroke was the third leading cause of death globally in 2021.^1^ In Japan, it ranked as the fourth leading cause of death in 2023, with approximately 100,000 deaths recorded.^2^ In 2020, Iwate Prefecture recorded the highest age-adjusted mortality rates for stroke in Japan, with rates of 147.2 and 84.3 per 100,000 for men and women, respectively, compared with the respective national rates of 93.8 and 56.4.^3^ Stroke is not only one of the leading causes of death but also a major contributor to long-term care dependency following onset.^4^ Reducing the incidence of stroke is a critical challenge at individual and societal levels.

The Iwate Tohoku Medical Megabank Organization (IMM) and Tohoku Medical Megabank Organization (ToMMo) were established under the Tohoku Medical Megabank Project (TMM Project) in response to the Great East Japan Earthquake in 2011 to provide medical assistance and improve health in areas severely affected by the earthquake. Within the project, genomic cohorts^5^^–^^8^ and a biobank^9^ were established for medical research, aiming to promote precision preventive medicine. As part of this initiative, we are developing personalized healthcare based on individual genetic information. To date, we have studied the impacts of reporting genomic results on individuals’ health management for monogenic diseases, such as hereditary cancers,^10^^–^^13^ a feasible setting supported by accumulating evidence on variant pathogenicity. More recently, for polygenic diseases, such as cardiovascular disease, expectations toward clinical application have increased with advances in developing polygenic score (PGS) models with enhanced accuracy.^14^^–^^16^

Based on the genomic data of domestic cohort studies, including data from a subset of the TMM Project cohorts, PGS models for ischemic stroke targeting Japanese and East Asian populations have been developed.^17^^–^^19^ Hachiya et al showed that the hazard ratio for developing ischemic stroke increased with the number of non-genetic risk factors (hypertension, diabetes, and smoking), both in the lower PGS group (first and second quintiles) and in the higher PGS group (third to fifth quintiles), based on a prospective cohort study.^18^ Notably, they also demonstrated that the hazard ratio of the subgroup without non-genetic risk factors in the higher PGS group was not significantly different from that in the lower PGS group.^18^ The results suggest that reducing non-genetic risk factors, in other words modifiable risk factors, would help to lower the risk of developing ischemic stroke, even in individuals with high genomic risk.

One common approach to reducing non-genetic risks is to inform individuals about their disease risk to encourage behavioral changes. Although genetic risk information is expected to motivate individuals to adopt health-promoting behaviors, previous studies have not yielded consistent results supporting this expectation. A meta-analysis reported little or no significant effect of genetic risk information on behavior.^20^ On the other hand, some studies have reported that informing participants about their polygenic risk for cardiovascular diseases, such as coronary heart disease or atherosclerotic cardiovascular disease, can lead to healthcare-related behavioral changes. These changes include seeking medical care,^21^^,^^22^ inquiring about the disease,^23^ and enhancing perceived personal control.^24^ However, to date, no studies have specifically investigated the effects of disclosing the polygenic risk for stroke. We hypothesized that awareness of one’s polygenic risk for ischemic stroke may cause behavioral changes aimed at reducing non-genetic risk factors for the disease.

PGS has already been commercialized for certain diseases, such as cancer and heart disease; however, its clinical application in various common diseases remains limited. Challenges to the clinical use of PGS include insufficient evidence from prospective studies, the need for risk communication strategies, and the consideration of ethical, legal, and social issues.^25^^–^^28^

In this study, we established a cohort in Iwate Prefecture using a randomized controlled design to investigate how disclosing PGS risk for ischemic stroke impacts health behaviors among working-age adults. The study is being conducted under the biobank framework of the TMM project.

## METHODS

This study is a randomized controlled trial regarding effects of knowing genetic risk for ischemic stroke; it is also a biobanking project. Participants’ biospecimens and health information were de-identified and stored securely in the TMM integrated biobank.^9^^,^^29^^,^^30^

### Participants

#### Design, inclusion criteria, and exclusion criteria

In April 2023, we started a cohort study targeting working-age individuals in Iwate Prefecture. The inclusion criteria were i) age ≥18 years, ii) affiliation with private or public organizations in Iwate Prefecture, iii) wishing to know their genomic risk for multifactorial diseases, and iv) non-participants of the TMM Community-Based Cohort Study (TMM CommCohort Study)^5^ and the TMM Birth and Three-Generation Cohort Study (TMM BirThree Cohort Study).^8^

#### Recruitment

Recruitment and baseline survey were conducted at the health checkup venues of the participating organizations.

**Figure 1.**
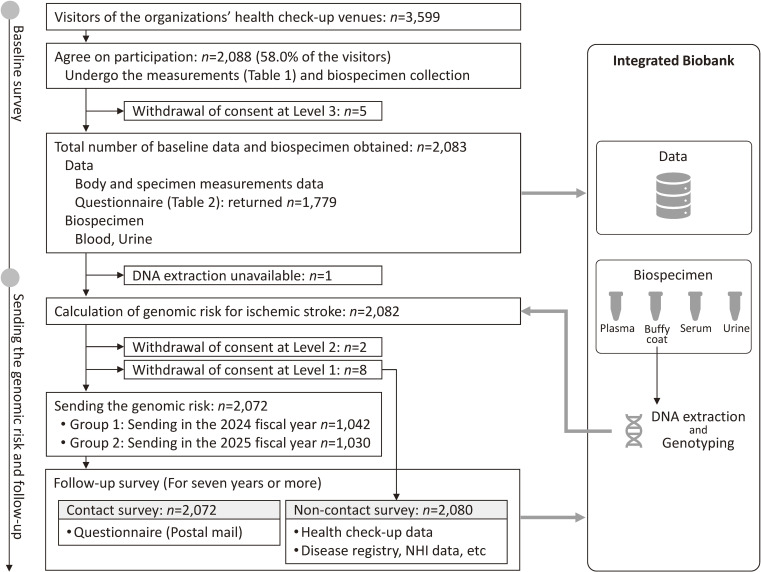
Outline of the Iwate PGS Assessment and Risk Communication Study. *Note*: Levels of withdrawal: Level 1, withdrawal from the contact survey; Level 2, withdrawal from all follow-up surveys; Level 3, withdrawal from all follow-up surveys and biobanking. PGS, polygenic score.

**Figure 2.**
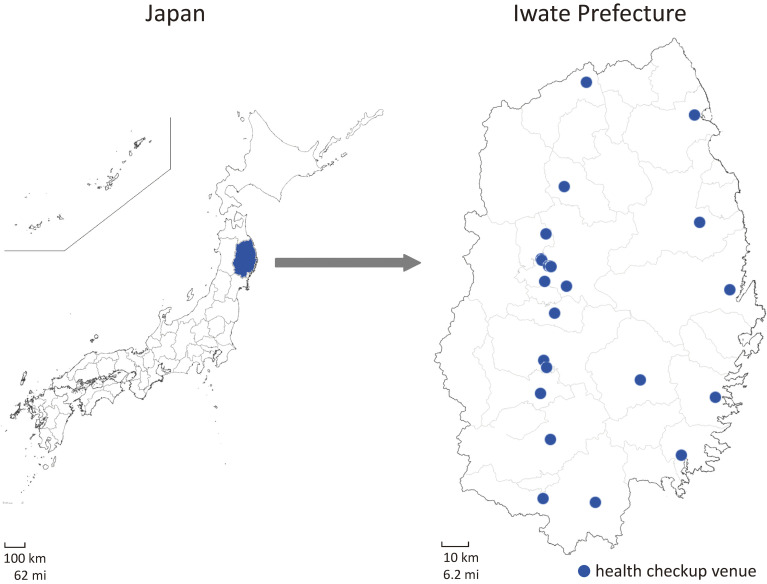
Location of Iwate Prefecture and health checkup venues

A flowchart of the study enrollment is shown in Figure 1. Before recruitment, brochures outlining the study were distributed to the employees. On the days of the health checkup, we visited the venues (Figure 2) and invited individuals to participate. Informed consent was obtained from each individual after a face-to-face explanation of the study. Participants underwent another blood and urine sampling in addition to that conducted for the health checkup. Similarly, they were given questionnaires and asked to complete and return them to IMM by post within approximately 2 weeks.

To appreciate their cooperation, a gift voucher worth 3,000 yen, as well as the results of blood and urine tests, nutritional intake status, and psychological status based on questionnaire responses, were sent within 2–4 months.

#### Sample size calculation

We calculated the required sample size to detect a between-group difference of 2.5 mm Hg in mean systolic blood pressure (SBP), assuming a standard deviation (SD) of 18.6 mm Hg.^31^ Based on a two-sided α of 0.05 and 80% power, along with a dropout rate of 10%, the calculated required sample size was 1,930 participants (965 per group).

#### Stratified randomization and risk reporting

Participants were randomly assigned in a 1:1 ratio to Group 1 or 2, stratified by age, sex, and workplace. Randomization was performed using R (version 4.0.3; the R Foundation for Statistical Computing, Vienna, Austria) to generate random numbers. Group 1 received the risk report in 2024, the year after enrollment, because the procedures to prepare the participants’ risk report were expected to take up to 1 year. Impacts will be assessed in 2025 using Group 2—in which participants have not yet received reports—as a contemporaneous control. For ethical considerations, Group 2 will receive risk reports after the 2025 survey.

The risk report for 2024, a five-page A4 booklet, was mailed to the participants’ homes. The PGS-based risk was presented as an odds ratio relative to the population average. The report included a colored image illustrating the individual’s risk for ischemic stroke within the overall population distribution, along with explanatory text about the risk and the disease.

### Outcomes and follow-up

#### Primary outcome measures

The primary outcome is the effect of receiving PGS risk information, observed over 2 years. Outcomes include body mass index (BMI) and stroke risk factors (blood pressure, blood glucose levels, blood lipids, atrial fibrillation, smoking habits, and alcohol consumption). Psychological impacts will be assessed using scales for mood (Profile of Mood States 2nd Edition [POMS2]^32^), anxiety (Kessler 6-item Psychological Distress Scale [K6]^33^), trauma (Impact of Event Scale-Revised [IES-R]^34^), and health awareness (Patient Activation Measure [PAM]^35^).

#### Follow-up survey

**Figure 3.**
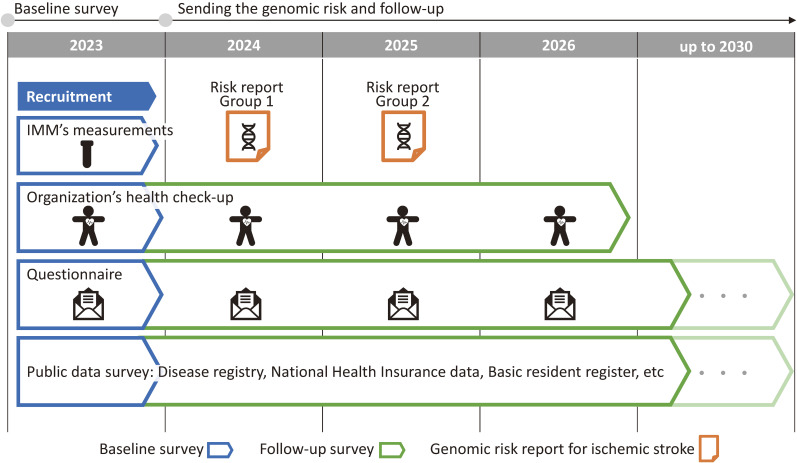
Time course of the Iwate PGS Assessment and Risk Communication Study. PGS, polygenic score.

The participants’ health checkup data will be obtained from the health checkup provider for 4 years (Figure 3). Additional data collection includes an annual questionnaire by postal mail, reviewing Iwate Prefecture disease registries (for stroke, heart disease, and cancer), reviewing National Health Insurance data, and confirming addresses or living status via the basic resident register of the respective municipalities for those who do not return the mail survey.

### Measurements

#### Blood and urine

Blood and urine samples were collected as detailed in Table 1. Blood collection (41 mL total) included 7 mL for organization health checkups (blood count, sugar tests, serum) and 34 mL for IMM measurements and biobanking. For IMM measurements, we used two serum tubes (9 mL each, VP-H050K; TERUMO Corp, Tokyo, Japan). For biobanking, we used one EDTA2Na tube (7 mL, VP-NA070K; TERUMO Corp.) for genomic DNA, and one serum tube (9 mL, VP-AS109K; TERUMO Corp.) for blood materials. Urine was collected in two tubes: one for health checkup measurements and another for IMM measurements and biobanking.

**Table 1.** Details of blood and urine tests during health checkups by organization and IMM

Measurement list	Organization’s health checkup	Additional measurements by IMM
**Biometric Tests**		
Body measurements		
Height	○	
Weight	○	
Abdominal circumference	○	
Body mass index	○	
Blood pressure and heart rate		
Systolic blood pressure	○	
Diastolic blood pressure	○	
Heart rate	○	
**Specimen Tests**		
Blood tests		
Red blood cell count	○	
Hemoglobin level	○	
Hematocrit value	○	
White blood cell count	○	
Platelet count	○	
White blood cell differential	○	
Total cholesterol	○	
Low-density lipoprotein cholesterol (LDL-C)	○	
High-density lipoprotein cholesterol (HDL-C)	○	
Triglycerides (TG)	○	
Aspartate aminotransferase (AST)	○	
Alanine aminotransferase (ALT)	○	
Gamma-glutamyl transferase (GGT)	○	
Fasting blood sugar	○	
Random blood sugar	○	
Hemoglobin A1c (HbA1c)	○	
Uric acid	○	
Blood urea nitrogen (BUN)	○	
Creatinine	○	
Serum creatinine test (eGFR)	○	
Glycated albumin		○
N-terminal pro–B-type natriuretic peptide (NT-pro BNP)		○
Cystatin C		○
eGFR (Cystatin)		○
High-sensitivity cardiac troponin T		○
Calcium		○
Phosphorus		○
Parathyroid hormone		○
Bone-specific alkaline phosphatase (BAP/CLEI)		○
Type I procollagen N-terminal propeptide (T-P1NP)		○
Type I collagen cross-linked N-telopeptide (Serum NTX)		○
Total protein		○
Albumin		○
Urine tests		
Urine glucose	○	
Urine protein	○	
Urine occult blood	○	
Urinary sodium		○
Urinary potassium		○
Urinary chloride		○
Urinary albumin		○
**Physiological Function Tests** ^a^		
Electrocardiogram	○	
Fundus examination	○	

IMM, Iwate Tohoku Medical Megabank Organization.

^a^Conducted if applicable.

#### Questionnaires

Baseline survey questionnaire items are listed in Table 2. In addition to the TMM CommCohort Study items described by Hozawa, Tanno, et al,^6^ we included assessments as follows: PAM^35^ for health awareness, POMS2,^32^ Genomic Knowledge Measure in the International Genetics Literacy and Attitudes Survey (iGLAS-GK),^36^ and an IMM-created coronavirus disease 2019 impact questions. The follow-up questionnaire will incorporate the IES-R^34^ and items assessing the participants’ perception of their PGS risk results.

**Table 2.** Details of the questionnaires at the baseline survey

Measurement	Measurement lists
Health consciousness	Understanding of health status awareness (Patient Activation Measure: PAM^a^)
Mood of the past week	Assessment of mood over the past week (Profile of Mood States 2nd Edition: POMS2^b^)
Knowledge of genetics	Genomic Knowledge Measure in the International Genetics Literacy and Attitudes Survey (iGLAS-GK^c^)
Basic information	Current height, weight (current, at the past year, and at birth), parents and grandparents’ birth year, year of death, place of birth, and current residence
Physical activities	Presence of a busier season involving higher physical activity than usual in the past year, frequency of physical activities and hours spent per session in the past year (commuting, work, and household chores), frequency of physical activities and hours spent per session in the past year (leisure time)
Alcohol drinking	Drinking status
Cigarette smoking	Smoking status and information on passive smoking
Outing and daily activities	Frequency of going out, transportation and surroundings
Psychological stress	Self-reported stress, Kessler Psychological Distress Scale (K6^d^)
Medications, dietary supplements, and health foods	Information on drugs and regularly used supplements
Family structure	Marital status, numbers of children and family members, cohabitants, caring for the family
Family history	Family and own history of diseases up to second-degree relatives
Health condition	Treatment status (hypertension, diabetes, hyperlipidemia, and dental care), self-reported health condition, usage of information and communication devices, difficulty with daily physical activities, habits for maintaining health, situation and level of satisfaction with social activities, experience with general anesthesia, health-related QOL (EQ-5D-3L^e^), health condition on the day of answering the questionnaire, issues with sense of taste, and influenza vaccination.
Impacts of COVID-19	Changes in work, life, relationships, medical visits, and medications
Occupation	Employment status, classification of industries, and occupation
Sleep	Hours of sleep, Athens Insomnia Scale (AIS^f^), and information on sleep medication
Social network	Lubben social network scale (LSNS-6^g^) and social capital
Depressive symptoms	Center for epidemiologic studies depression scale (CES-D^h^)
Damage and memory of the Great East Japan Earthquake	Condition of damage to the house, trauma, and experiences of loss
Information on obstetrics and gynecology (woman only)	History of pregnancy and childbirth, menstruation, and hormone medication
Dietary information	Food frequency questionnaire (FFQ^i^) and several diet-related questions
Life events	Holmes and Rahe stress scale^j^
Trait of worry	Penn State Worry Questionnaire (PSWQ^k^)
Problematic alcohol use	The questions focus on Cutting down drinking, Annoyance by criticism, Guilty feelings, and Eye-openers (CAGE^l^)

COVID-19, coronavirus disease 2019.

Psychological and knowledge scales:

^a^PAM assesses engagement in health management. Two versions exist (10- and 13-item versions). We used the 13-item version (4-point Likert scale); higher scores reflect greater engagement.

^b^POMS2 measures six mood states (Tension–Anxiety, Depression–Dejection, Anger–Hostility, Vigor–Activity, Fatigue–Inertia, and Confusion–Bewilderment) and state of Friendliness. Two versions of the scale exist (65-item and 35-item versions); we used the 35-item version with a 5-point Likert scale.

^c^iGLAS-GK assesses genome literacy through 20 items with 2- or 4-option multiple-choice questions. Scores range from 0 to 20. The Japanese general adult population mean is 8.4.

^d^K6 assesses psychological distress using six items (5-point Likert scale; scores range 0–24). Cutoffs: ≥5 (distress), ≥9 (mood/anxiety disorder), ≥13 (severe mental disorder).

^e^EQ-5D-3L assesses the health-related quality of life (QOL). It consists of five items (mobility, self-care, usual activities, pain/discomfort, and anxiety/depression) with a 3-level option. The combination of responses to the five items is converted to a QOL score ranging from 0 (death) to 1 (perfect health).

^f^AIS is a scale for assessing insomnia symptoms. It consists of eight items, each with a 4-level option, yielding a total score of 0–24 points. Scores ≥6 suggest possible clinical insomnia.

^g^LSNS-6 is a scale for assessing social isolation. It consists of six items, each with a 6-level option, yielding a total score of 0–30 points. Scores <12 indicate the risk of social isolation.

^h^CES-D is a scale for assessing depressive symptoms. It consists of 20 items, each with a 4-point Likert scale, yielding a total score of 0–60 points. In the Japanese version, scores ≥16 indicate the risk of depression.

^i^FFQ estimates the intake of major nutrients and energy; no summary score is calculated.

^j^Holmes and Rahe Stress Scale assigns “life change units” (LCUs) to 43 events (eg, Spouse’s death = 100, change in eating habits = 15) in the past year. Total scores: 150–299 (moderate stress), ≥300 (high stress).

^k^PSWQ assesses the degree of chronic worry. It consists of 16 items with a 5-point Likert scale, yielding a total score of 16–80 points, and higher scores indicate a stronger tendency to worry.

^l^CAGE Questionnaire screens for alcohol dependence. It consists of four Yes/No items, and two or more “Yes” responses suggest possible alcohol dependence.

#### Calculation of the PGS

Genomic DNA from participants was extracted from peripheral blood mononuclear cells and genotyped using the Axiom Japonica Array NEO.^37^ For the quality control step, we removed variants that were non-autosomal, with call rates of <0.99, with minor allele frequencies of <0.01, and/or with *P*-values <1e-5 for the Hardy–Weinberg equilibrium test. We subsequently performed genotype imputation using the 3.5KJPNv2 reference panel of haplotypes,^38^ as previously described,^37^ in two separate batches: 1,614 individuals recruited in April–May 2023 and 468 recruited in August–November 2023. Based on the imputed genotype data, the PGS for ischemic stroke was calculated for each participant using the integrated PGS model for East Asians established in the international collaborative study GIGASTROKE (PGS002725).^19^ The calculated PGS was normalized and converted into percentiles with reference to the mean, SD, and distribution of the PGS of the TMM community-based cohort, which is a residential cohort in the overlapping region of the present study.^39^ The percentiles were converted to relative risks based on the odds ratios that had been evaluated in an independent Japanese biobank and reported as part of the GIGASTROKE study.^19^

### Ethical issues

#### Informed consent

Informed consent was obtained through face-to-face interviews by trained Genome Medical Research Coordinators of IMM to provide information on medical studies. As a crucial part of the explanation, we informed participants that the study involved assessing the PGS risk of diseases based on genomic information and that participants would receive the risk reports, follow-up surveys would be conducted, and their blood and urine samples would be stored in the TMM integrated biobank for use in medical research. The brochure distributed to employees before recruitment provided a general explanation of the risk factors for multifactorial diseases and the concept of disease prevention. Additionally, we created a publicly accessible video explaining the risks of developing multifactorial diseases available on the Web (https://www.youtube.com/watch?v=XFMahLZiBC8), allowing participants to refer to it at any time.

To further ensure voluntary participation, participants’ data, including ischemic stroke PGS results, were not shared with their employing organizations, preventing participants from receiving requests from these organizations regarding study participation.

#### Management of withdrawal of consent

Participants were offered three withdrawal options (Levels 1–3). Level 1 refused only mailed items, such as the risk report and questionnaires, while permitting follow-up of their public records, such as the basic resident register, and the use of biospecimens and data. Level 2 refused both mailings and follow-up surveys but permitted the use of biospecimens and data. Level 3 refused all surveys and required disposal of samples and data.

#### Support system

Our contact information was provided on all materials distributed to the participants to address their potential questions or concerns. Two geneticists and one certified genetic counselor were responsible for responding, particularly regarding PGS risks or genetic concerns. We anticipated that participants would consult their general practitioners. Consequently, the support system was available to physicians to provide explanatory support regarding the PGS.

#### Ethical Committee and Genome Cohort Cooperation Working Group

The Iwate PARC Study was approved by the Ethics Committee of Iwate Medical University (MH2022-131). The protocol was developed with guidance from the Return of Genomic Results Review Committee, which comprises Japanese experts in genomics and clinical genetics and oversees genetic information disclosure studies within the TMM Project.

### Biobanking

Biospecimens and measurement data were securely stored in the TMM integrated biobank following the established operation process.^9^^,^^29^^,^^30^ We collected three blood (plasma, buffy coat, and serum fractions) and urine samples. Measurement data (Table 1) and questionnaire responses (Table 2) were cleaned at IMM, then transferred to ToMMo for registration in the integrated biobank system.

## RESULTS

### Baseline characteristics

**Figure 4.**
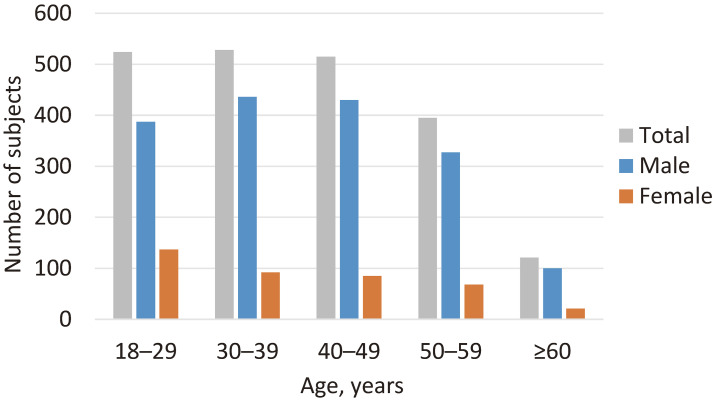
Age-sex distribution of participants (*n* = 2,083)

Recruitment and baseline survey were conducted between April 6 and November 13, 2023. The organizations were in the following industrial areas: one in the police sector, three in the manufacturing sector, and one in the hospitality sector. The health checkup venues covered almost all the inland and coastal areas of Iwate Prefecture (Figure 2). In total, 2,088 of the 3,599 individuals who visited these checkup venues consented to participate (58.0% overall consent rate, ranging from 28.7 to 64.1% in the different organizations; Table 3): 1,619 (79.8%) were from the prefectural police organization; 60 (3.0%), 113 (5.6%), and 260 (12.8%) were from the manufacturing industry; and 36 (1.8%) were from the hospitality industry. A total of 1,779 participants answered and returned the questionnaire (return rate: 85.2%). Excluding five participants who withdrew their consent at Level 3, as of May 28, 2024, the number of eligible participants was 2,083 (age range: 18–74 years, mean: 39.9; SD, 12.4 years), of which 1,680 were men (mean age: 40.3; SD, 12.3 years) and 403 were women (mean age: 37.9; SD, 12.9 years; Table 4 and Figure 4). Each of the 18–29-year, 30’s, and 40’s age groups included approximately 500 participants and constituted the main age range for both sexes. The largest proportion was men in their 30’s and women aged 18–29 years. Seven hundred eighty-two participants (37.5%) had a BMI of ≥25.0 kg/m^2^: 696 men (41.4%, ranging from 36.4–43.7% across age groups) and 86 women (21.3%, ranging from 11.7–33.3%). Two hundred thirty-one participants (11.1%) were classified as having an SBP ≥140 mm Hg: 211 men (12.6%, ranging from 5.7–29.0% across age groups) and 20 women (5.0%, ranging from 0.7–14.3%). A total of 438 (21.0%) and 1,115 (53.5%) participants were current smokers and drinkers, respectively: 416 (24.8%) and 954 (56.8%) for men; 22 (5.5%) and 161 (40.0%) for women. Regarding health history, 394 (18.9%), 136 (6.5%), 257 (12.3%), and 12 (0.6%) participants self-reported a history of hypertension, diabetes, hyperlipidemia, and ischemic stroke, respectively: 354 (21.1%), 131 (7.8%), 236 (14.0%), and 11 (0.7%) for men; 40 (9.9%), 5 (1.2%), 21 (5.2%), and 1 (0.2%) for women. Men had higher rates of these conditions than women for all four diseases. The mean score on the iGLAS-GK was 9.9 (SD, 2.9) out of 20 points: 9.7 (SD, 2.8) for men and 10.5 (SD, 3.0) for women.

**Table 3.** Number of participants who were recruited for and participated in the Iwate PARC Study

	Total	Organization				

		A	B	C	D	E
**Numbers**						
who were recruited (%)	3,599 (100.0)	2,525 (70.2)	95 (2.6)	394 (10.9)	479 (13.3)	106 (2.9)
who participated (%)	2,088 (100.0)	1,619 (79.8)	60 (3.0)	113 (5.6)	260 (12.8)	36 (1.8)
consent rate	58.0%	64.1%	63.2%	28.7%	54.3%	34.0%
who returned questionnaire (%)	1,779 (100.0)	1,377 (77.4)	55 (3.1)	104 (5.8)	218 (12.3)	25 (1.4)
return rate^a^	85.2%	85.1%	91.7%	92.0%	83.8%	69.4%
who withdraw the consent at Level 1–3	15	10	0	3	1	1
**Type of Industry**		Police sector	Manufacturing	Manufacturing	Manufacturing	Hospitality

PARC, PGS Assessment and Risk Communication.

^a^Proportion of questionnaire returns among participants.

**Table 4.** Baseline characteristics of eligible participants by age and sex

	Total	Male						Female					

		Sub-total	Organization					Sub-total	Organization				
	
			A	B	C	D	E		A	B	C	D	E
	*N* = 2,083	*N* = 1,680	*N* = 1,348	*N* = 40	*N* = 65	*N* = 207	*N* = 20	*N* = 403	*N* = 267	*N* = 20	*N* = 47	*N* = 53	*N* = 16
	*n* (%)	*n* (%)	*n* (%)	*n* (%)	*n* (%)	*n* (%)	*n* (%)	*n* (%)	*n* (%)	*n* (%)	*n* (%)	*n* (%)	*n* (%)
**Age, years**													
18–29	524 (25.2)	387 (23.0)	338 (25.1)	3 (7.5)	5 (7.7)	39 (18.8)	2 (10.0)	137 (34.0)	102 (38.2)	8 (40.0)	10 (21.3)	12 (22.6)	5 (31.3)
30–39	528 (25.3)	436 (26.0)	355 (26.3)	3 (7.5)	8 (12.3)	67 (32.4)	3 (15.0)	92 (22.8)	62 (23.2)	5 (25.0)	13 (27.7)	10 (18.9)	2 (12.5)
40–49	515 (24.7)	430 (25.6)	329 (24.4)	10 (25.0)	18 (27.7)	68 (32.9)	5 (25.0)	85 (21.1)	56 (21.0)	4 (20.0)	8 (17.0)	14 (26.4)	3 (18.8)
50–59	395 (19.0)	327 (19.5)	266 (19.7)	18 (45.0)	16 (24.6)	22 (10.6)	5 (25.0)	68 (16.9)	42 (15.7)	3 (15.0)	9 (19.1)	12 (22.6)	2 (12.5)
≥60	121 (5.8)	100 (6.0)	60 (4.5)	6 (15.0)	18 (27.7)	11 (5.3)	5 (25.0)	21 (5.2)	5 (1.9)	0 (0.0)	7 (14.9)	5 (9.4)	4 (25.0)
	Mean age (SD)												
	39.9 (12.4)	40.3 (12.3)	39.6 (12.2)	50.2 (10.7)	49.7 (12.2)	39.3 (10.7)	48.7 (13.3)	37.9 (12.9)	36.1 (11.9)	35.5 (10.9)	42.4 (14.1)	42.6 (12.4)	43.2 (20.1)
**BMI, kg/m^2^**													
<18.5	86 (4.1)	37 (2.2)	19 (1.4)	2 (5.0)	6 (9.2)	9 (4.3)	1 (5.0)	49 (12.2)	23 (8.6)	3 (15.0)	7 (14.9)	12 (22.6)	4 (25.0)
18.5 to <25.0	1,207 (57.9)	947 (56.4)	752 (55.8)	23 (57.5)	34 (52.3)	127 (61.4)	11 (55.0)	260 (64.5)	186 (69.7)	14 (70.0)	23 (48.9)	26 (49.1)	11 (68.8)
≥25.0	782 (37.5)	696 (41.4)	577 (42.8)	15 (37.5)	25 (38.5)	71 (34.3)	8 (40.0)	86 (21.3)	51 (19.1)	2 (10.0)	17 (36.2)	15 (28.3)	1 (6.3)
NA (pregnant women)	8 (0.4)	—	—	—	—	—	—	8 (2.0)	7 (2.6)	1 (5.0)	0 (0.0)	0 (0.0)	0 (0.0)
**SBP, mm Hg**													
<140	1,852 (88.9)	1,469 (87.4)	1,181 (87.6)	35 (87.5)	57 (87.7)	184 (88.9)	12 (60.0)	383 (95.0)	255 (95.5)	20 (100.0)	43 (91.5)	50 (94.3)	15 (93.8)
≥140	231 (11.1)	211 (12.6)	167 (12.4)	5 (12.5)	8 (12.3)	23 (11.1)	8 (40.0)	20 (5.0)	12 (4.5)	0 (.0)	4 (8.5)	3 (5.7)	1 (6.3)
**Smoking status**													
Non-smoker	921 (44.2)	617 (36.7)	498 (36.9)	14 (35.0)	20 (30.8)	82 (39.6)	3 (15.0)	304 (75.4)	214 (80.1)	15 (75.0)	35 (74.5)	33 (62.3)	7 (43.8)
Ex-smoker	416 (20.0)	391 (23.3)	321 (23.8)	9 (22.5)	20 (30.8)	38 (18.4)	3 (15.0)	25 (6.2)	12 (4.5)	1 (5.0)	5 (10.6)	5 (9.4)	2 (12.5)
Current smoker	438 (21.0)	416 (24.8)	318 (23.6)	16 (40.0)	20 (30.8)	53 (25.6)	9 (45.0)	22 (5.5)	12 (4.5)	0 (0.0)	4 (8.5)	6 (11.3)	0 (0.0)
Missing	308 (14.8)	256 (15.2)	211 (15.7)	1 (2.5)	5 (7.7)	34 (16.4)	5 (25.0)	52 (12.9)	29 (10.9)	4 (20.0)	3 (6.4)	9 (17.0)	7 (43.8)
**Alcohol use**													
Constitutionally never drinker	87 (4.2)	66 (3.9)	48 (3.6)	4 (10.0)	2 (3.1)	12 (5.8)	0 (0.0)	21 (5.2)	16 (6.0)	0 (0.0)	2 (4.3)	2 (3.8)	1 (6.3)
Never drinker	530 (25.4)	373 (22.2)	289 (21.4)	9 (22.5)	17 (26.2)	55 (26.6)	3 (15.0)	157 (39.0)	96 (36.0)	5 (25.0)	22 (46.8)	29 (54.7)	5 (31.3)
Past drinker	41 (2.0)	30 (1.8)	23 (1.7)	0 (0.0)	1 (1.5)	5 (2.4)	1 (5.0)	11 (2.7)	4 (1.5)	1 (5.0)	3 (6.4)	1 (1.9)	2 (12.5)
Current drinker	1,115 (53.5)	954 (56.8)	775 (57.5)	26 (65.0)	40 (61.5)	102 (49.3)	11 (55.0)	161 (40.0)	121 (45.3)	10 (50.0)	17 (36.2)	12 (22.6)	1 (6.3)
Missing	310 (14.9)	257 (15.3)	213 (15.8)	1 (2.5)	5 (7.7)	33 (15.9)	5 (25.0)	53 (13.2)	30 (11.2)	4 (20.0)	3 (6.4)	9 (17.0)	7 (43.8)
**Medical history**													
**Hypertension**													
History of the disease	394 (18.9)	354 (21.1)	272 (20.2)	15 (37.5)	26 (40.0)	33 (15.9)	8 (40.0)	40 (9.9)	24 (9.0)	0 (0.0)	10 (21.3)	4 (7.5)	2 (12.5)
Without history	1,293 (62.1)	997 (59.3)	800 (59.3)	22 (55.0)	33 (50.8)	137 (66.2)	5 (25.0)	296 (73.4)	202 (75.7)	15 (75.0)	33 (70.2)	40 (75.5)	6 (37.5)
Missing	396 (19.0)	329 (19.6)	276 (20.5)	3 (7.5)	6 (9.2)	37 (17.9)	7 (35.0)	67 (16.6)	41 (15.4)	5 (25.0)	4 (8.5)	9 (17.0)	8 (50.0)
**Diabetes**													
History of the disease	136 (6.5)	131 (7.8)	99 (7.3)	5 (12.5)	11 (16.9)	12 (5.8)	4 (20.0)	5 (1.2)	3 (1.1)	0 (0.0)	0 (0.0)	1 (1.9)	1 (6.3)
Without history	1,532 (73.5)	1,204 (71.7)	961 (71.3)	30 (75.0)	47 (72.3)	157 (75.8)	9 (45.0)	328 (81.4)	221 (82.8)	15 (75.0)	42 (89.4)	43 (81.1)	7 (43.8)
Missing	415 (19.9)	345 (20.5)	288 (21.4)	5 (12.5)	7 (10.8)	38 (18.4)	7 (35.0)	70 (17.4)	43 (16.1)	5 (25.0)	5 (10.6)	9 (17.0)	8 (50.0)
**Hyperlipidemia**													
History of the disease	257 (12.3)	236 (14.0)	188 (13.9)	10 (25.0)	16 (24.6)	19 (9.2)	3 (15.0)	21 (5.2)	17 (6.4)	0 (0.0)	1 (2.1)	2 (3.8)	1 (6.3)
Without history	1,422 (68.3)	1,107 (65.9)	878 (65.1)	27 (67.5)	41 (63.1)	151 (72.9)	10 (50.0)	315 (78.2)	210 (78.7)	15 (75.0)	41 (87.2)	42 (79.2)	7 (43.8)
Missing	404 (19.4)	337 (20.1)	282 (20.9)	3 (7.5)	8 (12.3)	37 (17.9)	7 (35.0)	67 (16.6)	40 (15.0)	5 (25.0)	5 (10.6)	9 (17.0)	8 (50.0)
**Atrial fibrillation**													
History of the disease	15 (0.7)	15 (0.9)	13 (1.0)	0 (0.0)	2 (3.1)	0 (0.0)	0 (0.0)	0 (0)	0 (0.0)	0 (0.0)	0 (0.0)	0 (0.0)	0 (0.0)
Without history	1,765 (84.7)	1,414 (84.2)	1,126 (83.5)	39 (97.5)	58 (89.2)	174 (84.1)	17 (85.0)	351 (87.1)	238 (89.1)	16 (80.0)	44 (93.6)	44 (83.0)	9 (56.3)
Missing	303 (14.5)	251 (14.9)	209 (15.5)	1 (2.5)	5 (7.7)	33 (15.9)	3 (15.0)	52 (12.9)	29 (10.9)	4 (20.0)	3 (6.4)	9 (17.0)	7 (43.8)
**Ischemic stroke**													
History of the disease	12 (0.6)	11 (0.7)	10 (0.7)	0 (0.0)	0 (0.0)	1 (0.5)	0 (0.0)	1 (0.2)	0 (0.0)	0 (0.0)	1 (2.1)	0 (0.0)	0 (0.0)
Without history	1,768 (84.9)	1,418 (84.4)	1,129 (83.8)	39 (97.5)	60 (92.3)	173 (83.6)	17 (85.0)	350 (86.8)	238 (89.1)	16 (80.0)	43 (91.5)	44 (83.0)	9 (56.3)
Missing	303 (14.5)	251 (14.9)	209 (15.5)	1 (2.5)	5 (7.7)	33 (15.9)	3 (15.0)	52 (12.9)	29 (10.9)	4 (20.0)	3 (6.4)	9 (17.0)	7 (43.8)
**Psychological or knowledge measurements**													
**K6**													
0–4	1,057 (50.7)	877 (52.2)	763 (56.6)	19 (47.5)	24 (36.9)	65 (31.4)	6 (30.0)	180 (44.7)	135 (50.6)	5 (25.0)	23 (48.9)	13 (24.5)	4 (25.0)
5–8	337 (16.2)	267 (15.9)	197 (14.6)	7 (17.5)	19 (29.2)	39 (18.8)	5 (25.0)	70 (17.4)	45 (16.9)	3 (15.0)	8 (17.0)	10 (18.9)	4 (25.0)
9–12	223 (10.7)	164 (9.8)	109 (8.1)	7 (17.5)	7 (10.8)	40 (19.3)	1 (5.0)	59 (14.6)	35 (13.1)	3 (15.0)	7 (14.9)	13 (24.5)	1 (6.3)
13–24	157 (7.5)	115 (6.8)	66 (4.9)	6 (15.0)	10 (15.4)	30 (14.5)	3 (15.0)	42 (10.4)	23 (8.6)	5 (25.0)	6 (12.8)	8 (15.1)	0 (0.0)
Missing	309 (14.8)	257 (15.3)	213 (15.8)	1 (2.5)	5 (7.7)	33 (15.9)	5 (25.0)	52 (12.9)	29 (10.9)	4 (20.0)	3 (6.4)	9 (17.0)	7 (43.8)
	Mean score (SD)												
	4.9 (5.1)	4.6 (5.0)	3.9 (4.6)	6.3 (6.0)	6.6 (5.4)	7.4 (5.9)	7.5 (5.2)	6.0 (5.5)	5.4 (5.2)	8.6 (5.5)	6.3 (6.4)	8.3 (6.0)	4.8 (3.5)
**iGLAS-GK**													
0–5	60 (2.9)	50 (3.0)	42 (3.1)	1 (2.5)	1 (1.5)	6 (2.9)	0 (0.0)	10 (2.5)	7 (2.6)	1 (5.0)	1 (2.1)	1 (1.9)	0 (0.0)
6–10	893 (42.9)	752 (44.8)	621 (46.1)	13 (32.5)	21 (32.3)	87 (42.0)	10 (50.0)	141 (35.0)	94 (35.2)	8 (40.0)	17 (36.2)	18 (34.0)	4 (25.0)
11–15	519 (24.9)	404 (24.0)	324 (24.0)	13 (32.5)	17 (26.2)	48 (23.2)	2 (10.0)	115 (28.5)	84 (31.5)	4 (20.0)	12 (25.5)	11 (20.8)	4 (25.0)
16–20	69 (3.3)	50 (3.0)	41 (3.0)	3 (7.5)	2 (3.1)	4 (1.9)	0 (0.0)	19 (4.7)	17 (6.4)	0 (0.0)	2 (4.3)	0 (0.0)	0 (0.0)
Missing	542 (26.0)	424 (25.2)	320 (23.7)	10 (25.0)	24 (36.9)	62 (30.0)	8 (40.0)	118 (29.3)	65 (24.3)	7 (35.0)	15 (31.9)	23 (43.4)	8 (50.0)
	Mean score (SD)												
	9.9 (2.9)	9.7 (2.8)	9.7 (2.8)	11.0 (3.2)	10.4 (2.9)	9.5 (2.8)	8.5 (1.6)	10.5 (3.0)	10.6 (3.1)	10.2 (2.6)	10.6 (3.2)	9.8 (2.3)	10.5 (1.5)

iGLAS-GK, Genomic Knowledge Measure in the International Genetics Literacy and Attitudes Survey; K6, Kessler Psychological Distress Scale; NA, not available; SBP, systolic blood pressure; SD, standard deviation.

Excluding the 15 participants who withdrew their consent at any level and one participant without an available DNA sample, 2,072 of the 2,088 participants remained eligible to receive PGS risk and full follow-up surveys. The distribution of the ischemic stroke PGS risk among the 2,072 individuals included in this study is shown in Table 5. Two hundred participants (9.7%) had a PGS percentile between 45 and 55, indicating an average risk. A total of 984 participants (47.4%) had a risk greater than 1.4 times the average, of whom 57 (2.7%) had a risk exceeding 2.2 times the average. In addition, 888 participants (42.9%) had a lower-than-average risk. The PGS-based risk was reported in 2024 to Group 1—1,042 eligible participants—as the intervention group.

**Table 5.** Distribution of genomic risk for ischemic stroke and risk reported to participants

Percentile of iPGS	Risk reported to participants (-fold)	Number of participants *n* (%)
99.9–100	3.4	3 (0.1)
99.5 to <99.9	2.2	8 (0.4)
97.5 to <99.5	2.1	46 (2.2)
95 to <97.5	1.9	54 (2.6)
90 to <95	1.8	117 (5.6)
80 to <90	1.6	227 (11.0)
55 to <80	1.4	529 (25.5)
45 to <55 (reference)	1.0 (average)	200 (9.7)
0.1 to <45	<1.0	888 (42.9)

Total		2,072 (100)

iPGS, integrative polygenic risk score.

### Biobanking and genotyping

Regarding data and biospecimen storage, excluding the five participants who withdrew at Level 3, 2,083 participants’ data and biospecimens were securely stored in the TMM integrated biobank (Figure 1). All samples and data were de-identified in multiple steps. At each step, correspondence tables linking previous and new identifications (IDs) were created, enabling re-identification when returning risk results to participants. After excluding one participant without an available DNA sample, we performed genotyping of 2,082 participants to calculate the PGS for ischemic stroke. The genotyping data also stored in the biobank.

## DISCUSSION

### Participant characteristics

Approximately 2,000 workers in Iwate Prefecture were enrolled in this study in 2023. The majority of participants were aged between 18 and 49 years, resulting in a relatively young study population compared to the Japanese working population.^40^

The 2023 National Health and Nutrition Survey (NHNS) reported that 23.2–35.0% of men and 11.8–25.0% of women aged between 20 and 69 years had a BMI ≥25.0 kg/m^2^,^41^ against which our participants exhibited similar or higher proportions across all age groups. Regarding SBP, the NHNS reported that 2.1–29.2% of men and 0.0–26.9% of women had an SBP ≥140 mm Hg.^42^ Our participants showed both higher and lower rates, with no consistent pattern by sex or age.

There was a notable imbalance in sex distribution, with approximately 80% of participants being male. Although demographic data for non-participants were unavailable, interviews with occupational health staff suggested that the imbalance reflects the overall workforce composition of the five organizations. Similarly, the high number of police workers, followed by those in manufacturing and hospitality, likely represents the overall number of workers across these organizations. Thus, bias related to sex and workplace is likely minimal, though it may still be present.

The mean iGLAS-GK score was 9.9 (SD, 2.9). In a previous study, the mean iGLAS-GK score for general adults in Japan was 8.4 (SD, 2.6).^36^ This discrepancy, if statistically significant, may be attributed to: (i) methodological differences, such as use of a paper-based questionnaire in this study compared to a web-based questionnaire in the previous study; or (ii) higher genetic interest among our participants, possibly linked to the motivation to join the study.

Twelve participants (0.6% of the cohort) self-reported a history of ischemic stroke (Table 4). Although this number is too small to affect group-level statistics, we recognize that prior stroke could influence risk perception and health behaviors. Therefore, these individuals will be excluded from the primary outcome analyses. They will nonetheless be retained in the baseline summary and followed longitudinally as an exploratory subgroup to gain insights into secondary-prevention behaviors for the disease.

### Risk reporting

The primary outcome will be observed after 1 year via risk reporting. We chose this interval to allow time for stable behavioral change, based on previous studies reporting behavioral or psychological changes after 6 to 18 months.^21^^–^^24^ The risk report presents the individual risk by an odds ratio, colored graphics, and explanatory text. Instead of face-to-face counseling, the report is designed using numerical, graphical, and textual elements to promote autonomous risk understanding through multiple comprehension pathways.

We hypothesize that participants who receive an above-average PGS risk report will show improvements in stroke-related, non-genetic risk factors, an increase in health awareness scores (PAM^35^), and limited anxiety or distress (POMS2,^32^ K6,^33^ IES-R^34^).

### Strengths and limitations

#### Strengths

The main strengths of the Iwate PARC Study are as follows. First, this is the first large-scale PGS risk communication study on stroke in Japan. Several large-scale studies involving more than 1,000 participants on the clinical use of PGS have been conducted in other countries, mainly focusing on cardiovascular diseases.^21^^,^^43^^–^^45^ In these studies, PGS-based risk has been reported to participants along with educational materials about the disease and information comparing PGS to traditional risk factors. These studies showed satisfaction among both healthcare providers and participants, as well as behavioral changes, such as increased visits to medical institutions and adjustments to health management plans. To date, however, reports evaluating the use of PGS for stroke in individuals are lacking.

Second, a total of 2,073 participants were eligible, exceeding the planned sample size of 1,930. This sample size permits detection of a between-group difference in SBP of 2.5 mm Hg. This difference is clinically relevant: a prior study has indicated that a 2-mm Hg reduction in SBP is associated with a decrease in stroke mortality among adults of approximately 10%.^46^ This robust sample size thus strengthens the study design by ensuring adequate power to detect meaningful differences in SBP outcomes.

Third, more than half of the participants were aged <40 years. This age distribution provides comprehensive insights into the practical applications of PGS for preventive medicine. The younger generation, as well as the middle-aged and older generations, is a significant target for preventive medicine because the individuals would have the potential to effectively lower disease risk by earning health consciousness early in life. Based on these strengths, the study will provide a novel and valuable platform for evaluating PGS risk communication for ischemic stroke prevention in Japan.

Finally, the participants’ biospecimens and health information have been securely stored in the TMM integrated biobank and will be available for future medical research. The basic protocol of this study—biological measurements, questionnaire items, and genotyping method—is aligned with those used in previous cohorts of the TMM project.^5^^,^^8^ The project offers well-characterized samples and data of the general population (healthy or presymptomatic individuals). The Iwate PARC Study will complement the project and serve as a resource for forthcoming investigations to advance precision preventive medicine.

#### Limitations

This study has some limitations. First, the results may not be applicable to other regions of Japan or other populations, as it was conducted in Iwate Prefecture.

Second, there are sex and occupation imbalances: approximately 80% of the participants were male, and 80% were employees of the police sector. To address these imbalances, stratification and adjustment for sex and occupation will be performed in the outcome analyses, such as analysis of covariance and logistic regression. In addition, there may be a sampling bias by genomic literacy. The mean iGLAS-GK scores of the participants in this study were higher than those of the general Japanese adult reported in a previous study.^36^ However, this may reflect differences in the form of the questionnaire used (paper-based vs web-based; ie, mode effects). To evaluate potential mode effects, certain statistical analyses, such as multi-group confirmatory factor analysis and sensitivity analyses, will be performed.

Third, the incidence rate of stroke, including ischemic stroke, is <0.2% in Japan. For example, the age-adjusted incidence rates of cerebrovascular diseases per 100,000 person-years in the Japanese standard population were 176.8 and 97.0 in men and women, respectively, between 2013 and 2017 in Iwate Prefecture.^47^ With a sample size of approximately 2,000 individuals, observations regarding the impacts of PGS reporting on the onset of ischemic stroke, obtained in this study, would be inadequate.

### Conclusion

We initiated a study on the practice of PGS-based risk communication for ischemic stroke targeting more than 2,000 working-age individuals in Iwate Prefecture. The first evaluation of stroke risk factors and psychosocial status with respect to the impact of risk communication will be conducted 1 year after disclosure of the risk and will be followed up until 2030. This is the first large-scale PGS risk communication study on stroke in Japan. It is expected to provide essential initial insights that will contribute to the practice of PGS in personalized prevention medicine.
